# Prevalence of chronic hepatitis C infection in the general population: results from a national survey, Romania, 2020 to 2023

**DOI:** 10.2807/1560-7917.ES.2024.29.30.2300663

**Published:** 2024-07-25

**Authors:** Mira Hleyhel, Odette Popovici, Mihaela Leuștean, Suzanne Reed, Amal Sadou, Martina Furegato, Benjamin Bluemel, Erika Duffell, Otilia Mardh

**Affiliations:** 1 Cerner Enviza/Oracle Life Sciences, Paris, France; 2 National Institute of Public Health Romania - National Centre for Surveillance and Control of Communicable Diseases (NCSCCD), Bucharest, Romania; 3 National Institute of Public Health Romania - National Public Health Laboratory, Bucharest, Romania; 4 European Centre for Disease Prevention and Control, Stockholm, Sweden

**Keywords:** Hepatitis C, prevalence, general population, chronic infection, seroprevalence

## Abstract

**Introduction:**

A national study from 2006 to 2008 showed a high antibody prevalence of 3.2% against hepatitis C virus (HCV) in Romania, but more recent epidemiological data on hepatitis C prevalence are lacking.

**Aim:**

We aimed to estimate the current prevalence of HCV antibodies (anti-HCV) and chronic HCV infection in the general adult population in Romania, as a crucial element in monitoring progress towards eliminating hepatitis C.

**Methods:**

We used anonymised leftover sera from a SARS-CoV-2 survey conducted between July and October 2020 (n = 2,100), supplemented with sera collected prospectively between July 2022 and March 2023 (n = 574). These included sera collected from adults visiting laboratories for routine medical check-ups. Sera were tested for anti-HCV and HCV core antigen and classified according to anti-HCV and chronic infection status.

**Results:**

Of the total 2,674 specimens tested, 44 were anti-HCV-positive with a weighted anti-HCV prevalence of 1.4% (95% CI: 1.0–1.9), and 29 were HCV core antigen-positive with a weighted prevalence of chronic infection of 0.9% (95% CI: 0.5–1.2). The prevalence of chronic infection did not differ significantly between men and women. It was higher in persons 60 years and older (2.0%; 95% CI: 1.1–3.0) and in specimens from the North-East region (2.2%; 95% CI: 0.8–3.7).

**Conclusion:**

Although the overall HCV prevalence in Romania is currently low, targeted screening, prevention measures and treatment scale-up are needed especially for the population 60 years and older and in the north-eastern part of the country to achieve the goal of ending the hepatitis C epidemic.

**Key public health message**

**What did you want to address in this study and why?**

Up to date and accurate estimates of hepatitis C virus (HCV) infection are essential to guide public health interventions and monitor progress towards hepatitis C elimination targets. We decided to assess chronic HCV infection in the general population in Romania as the last national survey had been conducted 15 years previously, before diagnostic testing was scaled up and antiviral treatment became available.

**What have we learnt from this study?**

We tested 2,674 blood samples for antibodies against HCV - indicators of past infection, and for HCV core antigen - indicator of chronic infection. Our results indicate that the overall prevalence of chronic HCV infection has decreased in Romania since the previous study and is currently estimated at 0.9%. Chronic infection was most frequent among people ≥ 60 years (2.0%) and people from the North-East region of Romania (2.2%).

**What are the implications of your findings for public health?**

Romania is now a country with low frequency of hepatitis C, and the reduction in the prevalence over time suggests that the country is making progress towards hepatitis elimination. Our results highlighted two priority populations (those aged 60 years and older and those living in the North-East region of the country) for screening and intervention strategies.

## Introduction

Hepatitis C is an infectious disease caused by the hepatitis C virus (HCV) and is a major global public health problem with an estimated 50 million individuals chronically infected with the virus and 0.2 million deaths in 2022 [1]. Following acute infection with HCV, spontaneous clearance of the virus can occur, however 50–80% of patients subsequently develop chronic infection [2], which can lead to cirrhosis and hepatocellular carcinoma [3]. According to the World Health Organization (WHO), ca 1.5 million people are newly infected each year [4]. Risk factors associated with the spread of the infection include the sharing of injection equipment during injecting drug use [4], exposure to infected blood through inadequately sterilised medical equipment, transfusion of unscreened blood and blood products, and high-risk unprotected sexual behaviour [5].

The treatment of HCV has been revolutionised by the development of safe and well tolerated oral direct-acting antivirals (DAAs), which achieve sustained virologic response in more than 95% of HCV-infected patients [6]. Following the introduction of DAAs, the WHO has proposed the ending of hepatitis C as a public health threat by 2030 [7], a goal requiring multiple approaches including continued investment in primary prevention and substantially increased access to HCV testing and treatment [7].

Romania is a country in the south-east of Europe with a population of around 19 million in 2021 [8]. The most recent nationwide survey of HCV prevalence in Romania was conducted among the adult population (18–69 years) in the period 2006 to 2008 using serum specimens from 13,146 subjects who attended primary care [9]. This study found an overall estimated prevalence of antibodies to HCV (anti-HCV) of 3.2% (95% confidence interval (CI): 2.9–3.6). Two additional studies have been conducted more recently among the general population in Romania, but both were undertaken at the subnational level. These two studies also found elevated prevalence: one was conducted in 2008 and 2009 in the Sub-Carpathian and South-East regions of Romania (42% of the population), estimating anti-HCV prevalence to be 4.6% (95% CI: 3.8–5.5) [10]; the other was a study conducted in 2019 and 2020 in a single village in north-eastern Romania, reporting an anti-HCV prevalence of 2.6% (95% CI: 2.1–3.3) [11].

The prevalence of HCV has been identified by the WHO as a critical metric to monitor progress towards hepatitis elimination [12]. However, a systematic review conducted by the European Centre for Disease Prevention and Control (ECDC) and published in 2016 found that many countries in the European Union (EU) and European Economic Area (EEA) lacked up-to-date estimates of HCV prevalence and many surveys were conducted using a weak methodological design [13]. In this context, the ECDC developed the Sphere C protocol to assist EU/EEA countries in generating robust prevalence estimates and gain a better understanding of the epidemiology of HCV [14].

In light of the high HCV prevalence found in previous prevalence surveys and the lack of recent national epidemiological data on hepatitis C in Romania, the aim of our study was to estimate the seroprevalence of hepatitis C and the prevalence of chronic hepatitis C in the adult general population in Romania.

## Methods

### Study design and population

The study design was guided by recommendations of the Sphere C protocol [14]. One approach suggested in the Sphere C protocol is the retrospective testing of leftover sera from previous serosurveys that were based on a representative sample of the general population and included the collection of blood samples. This methodological approach was considered the most appropriate option for the current survey.

Our study used leftover sera from a previous cross-sectional survey conducted to assess the prevalence of SARS-CoV-2 antibody seropositivity in Romania using sera collected during regular medical check-ups in primary care [15]. The sampling consisted of two stages, with the first stage involving the selection of laboratories and the second stage the selection of individuals. The laboratories were selected based on specific criteria: they had to be handling a high volume of samples (more than 40,000 per year) and serving between July and October 2020 outpatients rather than patients requiring hospitalisation. Each of the 42 County Public Health Directorates across the country opted to include between three and five laboratories in the study, except for the Bucharest Public Health Directorate, which chose nine laboratories due to the large population size in the capital city. In the second stage, individuals of all age groups who attended for check-ups at the selected medical laboratories and who expressed their consent to participate were included based on systematic sampling (as described in [15]). Individuals with signs and symptoms of respiratory infection or who had requested COVID-19 testing were not included in the SARS-CoV-2 survey. Of leftover sera from the SARS-CoV-2 survey that were stored in the National Institute of Public Health in Bucharest, only sera corresponding to adults 18 years or older were included in the current HCV prevalence survey.

To compensate for the insufficient number of eligible samples available from the SARS-CoV-2 survey (due to insufficient volume of leftover sera and serum leakage), we conducted a prospective collection of new sera between July 2022 and March 2023 to complete the sample size and improve the representativeness of the sample in terms of age, sex and county/geographical distribution. For each county, we selected the laboratory with the highest capacity in terms of number of tests performed in 2021, followed by other laboratories in other cities in the county, in descending order of the number of tests performed in 2021. Sera were included from healthy adults aged 18 years or older visiting laboratories for routine medical check-ups of their health status and not for testing related to chronic HCV infection. To minimise bias in the prevalence estimate, laboratory personnel were trained by the County Public Health department epidemiologist to exclude specimens from patients diagnosed with hepato-biliary-pancreatic pathology or related signs and symptoms that attended for that specific pathology rather than for a routine medical check-up. Moreover, to minimise potential underestimation of the prevalence, we also excluded specimens from blood donors. Each selected laboratory identified specimens to be included through a systematic sampling procedure, using the same technique applied for the leftover sera from the SARS-CoV-2 study. To avoid duplicates between the two categories of serum samples (the ones originated from the COVID-19 seroprevalence study and those collected prospectively), we used the personal identifier to exclude duplicates, but we did not find any.

### Sample size

The sample size for each 10-year age group in each region was calculated using EpiInfo 7, version 7.2.3.1, based on an expected anti-HCV prevalence of 3% [16], for a 95% CI with an acceptable margin of error of 5% and a design effect of 1. After accounting for 5% losses, the estimated sample size was 47.5 in each age group in each region. Given that the calculation was based on seven 10-year age groups (18–29, 30–39, 40–49, 50–59, 60–69, 70–79 and ≥ 80 years) and eight regions, the total sample size for the study was 2,660.

### Laboratory testing algorithm and interpretation of results

Laboratory testing for HCV was performed at the National Institute of Public Health in Bucharest. The testing algorithm is summarised in the Figure. First, an enzyme immunoassay (ELISA) (reagent kits - 96-well microplate format, DIA PRO, Italy) was performed on all specimens to determine the presence of anti-HCV antibodies. The anti-HCV-positive sera were then tested for HCV-core antigen (Ag) using the Abbott chemiluminescence immunoassay (CLIA). Finally, anti-HCV results in HCV core Ag-negative specimens were tested using the anti-HCV line immunoblot assay (LIA) recomLine HCV IgG (Mikrogen Diagnostics, Neuried, Germany) in order to confirm their status. Specimens were classified as having resolved infection or chronic infection according to the algorithm outlined in the Figure.

**Figure:**
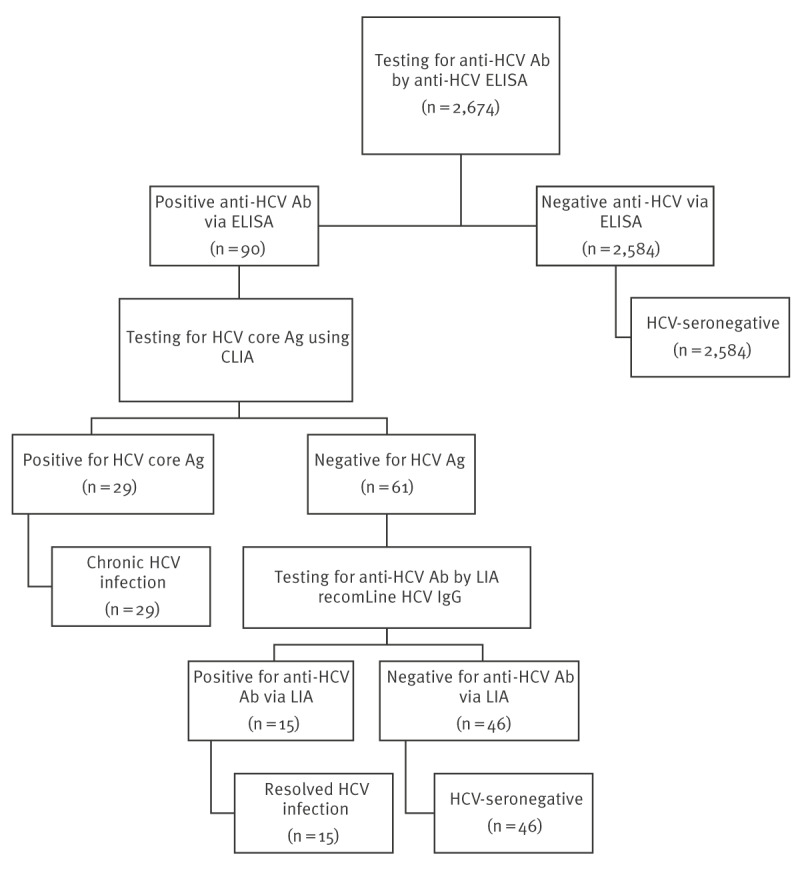
Hepatitis C virus testing algorithm and interpretation of test results, Romania, 2020–2023 (n = 2,674)

Ab: antibodies; Ag: antigen; CLIA: chemiluminescence immunoassay; ELISA: enzyme-linked immunosorbent assay; HCV: hepatitis C virus; LIA: line immunoblot assay.

### Statistical analysis

We calculated the anti-HCV prevalence and the prevalence of chronic HCV infection and their 95% CI overall and by age group, sex and region. To ensure that the prevalence estimates would be representative of the general population in Romania, the estimates were weighted for sex, age and region by applying the sampling fraction calculated from Romanian population data of 2020 [8].

We used chi-square test to compare the prevalence of chronic HCV infection and the seroprevalence between sex, age groups and regions. In addition, we performed pair-wise p value comparisons with Bonferroni multiple significance test correction to compare combinations of each age group pair and each region pair.

SAS version 9.4 was used for all the statistical analyses.

## Results

A total of 2,674 serum specimens were selected and tested from subjects throughout Romania: 2,100 specimens that had previously been collected for the SARS-CoV-2 survey [15] and 574 specimens that were collected prospectively.

Information on the demographic characteristics of the general population in Romania and of the sample before and after weighting is appended in the Supplementary material. The distribution of selected specimens in terms of sex and region was similar to the general Romanian population with the exception of individuals aged 30–59 years who were under-represented and of those 70 years and older who were over-represented.

The Figure illustrates the interpretation of HCV test results and specimen classification. Of the 2,674 specimens tested, 90 (3.4%) were found to be anti-HCV ELISA-positive and all 90 underwent subsequent HCV core Ag testing. Of these 90 specimens, 29 (32.2%) were considered to have chronic HCV infection confirmed by positive HCV core Ag, and of the 61 specimens that tested negative for the HCV core Ag, 15 (24.6%) were confirmed positive for anti-HCV by immunoblot assay indicating resolved infection. The remaining 46 specimens tested negative for anti-HCV by immunoblot assay and were considered seronegative.

After weighting for age, sex and population per region, the national anti-HCV prevalence was estimated to be 1.4% (95% CI: 1.0–1.9) (Table 1) and the prevalence of chronic HCV infection was 0.9% (95% CI: 0.5–1.2) (Table 2). No statistically significant sex differences were observed. When comparing by age group, the prevalences of anti-HCV and chronic HCV infection, respectively, were significantly higher in the age group 60 years and older (2.6%; 95% CI: 1.5–3.6 and 2.0%; 95% CI: 1.1–3.0) than in the age groups younger than 60 years (1.1%; 95% CI: 0.6–1.5 and 0.5%; 95% CI: 0.2–0.9).

**Table 1:** Weighted hepatitis C seroprevalence and estimated number of seropositive individuals, Romania, 2020–2023 (n = 2,674)

Demographic characteristics	Total weighted number of specimens in the study sample	Unweighted number of seropositive specimens in the study sample	Weighted number of seropositive specimens in the study sample	Weighted seroprevalence in Romania		Estimated number of seropositive individuals in Romania^a^	
				%	95% CI	n	95% CI
Overall	2,674	44	38.02	1.4	1.0–1.9	222,254	152,110–292,397
Sex							
Female	1,380.4	26	20.34	1.5	0.8–2.1	118,901	67,610–170,192
Male	1,293.6	18	17.68	1.4	0.7–2.0	103,352	55,506–151,197
Age groups (years)							
18–29	417.3	2	1.94	0.5	0.0–1.1	11,341	0–27,262
30–39	455.9	2	2.75	0.6	0.0–1.3	16,076	0–35,018
40–49	514.7	4	4.49	0.9	0.1–1.7	26,247	2,075–50,419
50–59	439.3	8	10.26	2.3	0.9–3.8	59,977	23,708–96,246
60–69	422.9	6	6.39	1.5	0.4–2.7	37,354	8,611–66,097
70–79	265.1	8	5.87	2.2	0.4–4.0	34,314	6,864–61,763
≥ 80	158.8	14	6.31	4.0	0.9–7.0	36,887	8,683–65,091
Age groups (years)							
< 60	1,827.2	16	19.49	1.1	0.6–1.5	113,932	63621–164,244
≥ 60	846.8	28	21.65	2.6	1.5–3.6	126,559	73,933–179,184
Region							
Bucuresti-Iflov	326.0	4	2.06	0.6	0.0–1.5	12,042	0–28,434
Center	317.1	1	0.36	0.1	0.0–0.5	2,104	0–8,975
North-East	425.0	13	12.23	2.9	1.3–4.5	71,493	32,005–110,981
North-West	351.9	2	1.83	0.5	0.0–1.3	10,698	0–26,157
South-East	328.7	4	3.54	1.1	0.0–2.2	20,694	0–42,134
South-Muntenia	405.4	7	7.55	1.9	0.6–3.2	44,135	12,947–75,323
South-West Oltenia	270.8	8	7.06	2.6	0.7–4.5	41,270	11,226–71,313
West	249.1	5	3.39	1.4	0.0–2.8	19,817	0–40,769

CI: confidence interval.

^a^ The number of seropositive individuals in Romania was calculated based on the prevalence estimates and the Romanian population data of 2020 [8].

**Table 2:** Weighted prevalence of chronic hepatitis C virus infection and estimated number of individuals with chronic hepatitis C virus infection, Romania, 2020–2023 (n = 2,674)

Demographic characteristics	Total weighted number of specimens in the study sample	Unweighted number of specimens with chronic HCV in the study sample	Weighted number of specimens with chronic HCV in the study sample	Weighted prevalence in Romania		Estimated number of individuals in Romania with chronic HCV^a^	
				%	95% CI	n	95% CI
Overall	2,674	29	23.43	0.9	0.5–1.2	136,965	81,749–192,182
Sex							
Female	1,380.4	17	13.09	0.9	0.4–1.5	76,520	35,263–117,776
Male	1,293.6	12	10.35	0.8	0.3–1.3	60,503	23,790–97,216
Age groups (years)							
18–29	417.3	2	1.94	0.5	0.0–1.1	11,341	0–27,262
30–39	455.9	1	1.41	0.3	0.0–0.8	8,242	0–21,826
40–49	514.7	1	0.96	0.2	0.0–0.6	5,612	0–16,827
50–59	439.3	4	4.89	1.1	0.1–2.1	28,586	3,390–53,781
60–69	422.9	4	4.61	1.1	0.1–2.1	26,949	2,483–51,415
70–79	265.1	6	4.52	1.7	0.2–3.3	26,422	2,272–50,572
≥ 80	158.8	11	5.11	3.2	0.5–6.0	29,872	4,392–55,352
Age groups (years)							
< 60	1,827.2	8	9.63	0.5	0.2–0.9	56,294	20,832–91,755
≥ 60	846.8	21	16.97	2.0	1.1–3.0	99,201	52,478–145,925
Region							
Bucuresti-Iflov	326.0	3	1.72	0.5	0.0–1.3	10,054	0–25,041
Center	317.1	1	0.36	0.1	0.0–0.5	2,104	0–8,975
North-East	425.0	11	9.53	2.2	0.8–3.7	55,710	20,738–90,681
North-West	351.9	2	1.83	0.5	0.0–1.3	10,698	0–26,157
South-East	328.7	2	1.48	0.5	0.0–1.2	8,652	0–22,559
South-Muntenia	405.4	4	3.95	1.0	0.0–1.9	23,091	430–45,751
South-West Oltenia	270.8	3	2.3	0.8	0.0–1.9	13,445	0–30,747
West	249.1	3	2.26	0.9	0.0–2.1	13,211	0–30,358

CI: confidence interval.

^a^ The number of chronically infected individuals in Romania was calculated based on the prevalence estimates and the Romanian population data of 2020 [8].

In regional comparisons, the highest anti-HCV prevalence was found in North-East (2.9%; 95% CI: 1.3–4.5), followed by South-West Oltenia (2.6%; 95% CI: 0.7–4.5), South-Muntenia (1.9%; 95% CI: 0.55–3.18) and West (1.36%; 95% CI: 0.00–2.80). A statistically significant difference in anti-HCV prevalence was observed between South-Muntenia (1.9%; 95% CI: 0.6–3.2) and Bucuresti-Iflov (0.6%; 95% CI: 0.0–1.5). No significant differences were observed for chronic HCV infection, with the highest prevalence observed in North-East (2.2%: 95% CI: 0.8–3.7), and the lowest prevalence observed in Center (0.1%; 95% CI: 0.0–0.5).

Based on the estimated weighted prevalence value, we estimated that there were 222,254 (95% CI: 152,110–292,397) individuals who were anti-HCV positive and 136,965 (95% CI: 81,749–192,182) chronically infected individuals among the general adult population in Romania. Individuals 60 years and older were estimated to represent 56.9% of those who were anti-HCV positive and 72.4% of those with chronic infection.

## Discussion

Our study provides updated epidemiological data on the burden of HCV infection in the general population in Romania. We found an estimated anti-HCV prevalence of 1.4% (95% CI: 1.0–1.9) and a prevalence of chronic HCV infection of 0.9% (95% CI: 0.5–1.2), corresponding to ca 137,000 chronically infected individuals, indicating Romania as a country of low HCV endemicity.

Our survey showed notable regional differences, with highest prevalence of HCV infection in the North-East region of Romania. This is consistent with the previous nationwide survey in Romania conducted in the period from 2006 to 2008, which identified the Moldavia region in the north-east of the country as the region with the highest prevalence of HCV in Romania [9]. A more recent study by Huiban et al. conducted between 2019 and 2020 in a village in the north-eastern region of the country reported an anti-HCV prevalence of 2.6% (95% CI: 2.1–3.1) [14]. Another study conducted in 2008 and 2009 in the South-East region of Romania and in the Sub-Carpathian region reported an anti-HCV prevalence rate of 4.6% (95% CI: 3.8–5.5) [13]. The South-East and North-East regions of Romania are areas that have a significant proportion of the population with low income and poor local public infrastructure and with limited access to healthcare, including access to testing and treatment of those with chronic infection [17]. In addition, a recent meta-analysis found that the prevalence of nosocomial infections is higher in low-income countries than in high-income countries, reflecting a lack of facilities and resources to implement optimal infection control programmes [18]. These factors may help explain the higher prevalence in these particular regions as these are factors that are likely to be associated with challenges to the effective implementation of strategies to end the hepatitis epidemic [9,11].

Previous studies in Romania [10,11,19,20] were conducted in specific populations such as hospitalised and haemodialysis patients or in specific regions, or were limited by methodological biases such as inadequate sampling and test accuracy. It is considered likely that they did not yield robust estimates of the national HCV prevalence among the general population. The previous national study in the general population in Romania, conducted from 2006 to 2008, reported an anti-HCV prevalence of 3.2% and HCV RNA prevalence of 3.0% [9]. Unlike that study, which used a multistage random cluster sampling to construct the study sample, our current study enrolled individuals who came for regular check-ups at medical laboratories and who were unlikely to be fully representative of the general adult population in Romania. To minimise potential selection bias, we selected specimens from laboratories in all regions of Romania and estimated weighted prevalence rates. Regional studies conducted in 2008 and 2009 and in 2019 and 2020 reported anti-HCV prevalence rates of 4.6% and 2.6%, respectively [9,11]. In general, despite the differences between the methodologies used in these studies, our findings suggest that the prevalence of HCV infection has declined over time. 

This observed decrease may be related to several factors but probably demonstrates the considerable efforts undertaken in recent years to eliminate and control the spread of HCV through scaling up of screening programmes that facilitated the diagnosis of infected individuals, the availability of antiviral treatment for those diagnosed, and increased rates of sustained virologic response achieved over time with the increased use of the highly effective DAAs [21]. In Romania, the data registered in the electronic platform of the National Health Insurance House showed that in May 2023, of the 44,147 patients enrolled in treatment until January 2023, 96.1% had sustained virologic response (data not shown: Odette Popovici, 17/05/2023, source: NHIH Romania). Indeed, the Romanian Ministry of Health has initiated a national programme called the National Framework Plan for the Control of Viral Hepatitis in Romania, 2019 to 2030 [22]. 

Given that the estimated number of seropositive individuals in the current study in Romania was 222,254 and the number of individuals with chronic hepatitis C was 136,965, and a total of 44,147 patients were treated until 2023, the chronicity rate would be approximately 80% ((136,965 + 44,147) / 222,254), with a spontaneous clearance rate of approximately 20%, rates similar to those reported in the literature. To our knowledge, HCV genotype was not found to be associated with spontaneous HCV clearance according to a published systematic review of longitudinal studies [23]. It should be noted that the circulating genotype in Romania is 1, with subtype 1b being the most common [24].

In addition, the lower prevalence found in our study could also be explained by a reduction in incidence of new infections through lower levels of transmission and exposure to risk factors [25,26], a low infection risk in younger age groups of the general population as well as by the mortality of the largest age cohorts of infected individuals as they progress to older age. In the study from 2006 to 2008, the prevalence of anti-HCV among cases aged 60–69 years was 6.5% [9], a population group that in our study had transitioned to age groups 70–79 and 80 years and older with a prevalence of 2.2% and 4.0%, respectively. The current age group 60–69 with an anti-HCV prevalence of 1.5% was in the age groups 40–49 and 50–59 in the previous study, with a prevalence of 2.7% and 3.7%, respectively. Even with a considerably lower prevalence, people 60 years and older account for the majority of hepatitis C cases (72.4% of cases identified in our study), indicating that they are a priority population to be targeted by follow-up measures (e.g. testing, treatment). It is noteworthy that high-risk populations, such as people who inject drugs, would not make a big contribution to the national estimates of the prevalence of HCV antibodies in Romania. According to the World Drug Report, the prevalence of injecting drug use in Romania was between 0.39% and 0.72% in 2017 [27].

In our study, while the weighted seroprevalence and prevalence of chronic HCV infection was slightly higher among women than among men, this difference was not statistically significant. This is different from the previous national study conducted between 2006 and 2008, which showed a significantly higher prevalence of HCV infection in females [9]. In that study, the investigators postulated that this could be attributed to an increased nosocomial risk experienced by females as a result of unsafe abortion practices because termination of pregnancies was illegal before 1990 when access to contraceptives was also very limited [19]. Most published HCV surveys from other European countries have either found no sex difference [28-30] or a higher prevalence in men [31], corresponding to injecting drug use, which is more common among men, as the main mode of transmission in most countries in Europe [25].

Our results showed that the oldest age group, 60 years and older, was the most affected by HCV infection. This finding is in line with the previous studies in Romania showing an increasing prevalence with age [9,11], and reflects high levels of nosocomial transmission before 1990 due to sub-optimal infection prevention and control resulting in a high risk of blood borne virus transmission through surgical procedures, receipt of blood transfusions or blood products from untested donors and/or receiving medical injections at home [9]. Another risk factor associated with increased prevalence of HCV identified in a previous study was having family members known to be HCV-positive, an effect of sharing of personal hygiene items [11]. 

Our study provides up-to-date national prevalence estimates that are representative of the general adult population in Romania, in regard to regions, age groups and sex, and includes weighting of the prevalence estimates. In addition, the use of leftover specimens collected for the SARS-CoV-2 seroprevalence study [15] allowed for timely and cost-effective estimation of HCV prevalence rates. However, it has several limitations. Specimen collection took place over two different time periods, one in 2020 for the SARS-CoV-2 survey and the other between 2022 and 2023 to complete the sample size and adjust its structure. Even though a screening programme has been in place in Romania since July 2021, as the proportion of newly collected samples for our study was small (21%), the impact on the prevalence rates from the second time period is likely to be low. Another limitation is related to the source population (i.e. those coming for medical checkups): the uninsured population (around 10%) could be under-represented. Another limitation is that the selection of laboratories was based on convenience sampling and was therefore not random. However, the laboratories were selected to represent all geographical regions in Romania, and the selection of samples from each laboratory was based on systematic random sampling, thus optimising age and sex representativeness. Finally, given the low prevalence rates observed in this study, for detecting significant differences between regions and to gain more statistical power, a larger sample size would have been needed, adding to the costs and logistics efforts.

The natural history of resolved/cured HCV infection is a decay in anti-HCV antibody titres over time and a proportion of anti-HCV-reactive, but LIA-negative, sera probably reflects this. This is not discussed in the Sphere C protocol, but this proportion will increase over time in future surveys, as the number of cured patients increase. In our opinion, this should be taken into account in the future studies guided by the same protocol.

Our results indicate a substantial decrease in the prevalence of HCV since the last nationwide survey in 2008 [9]. The highest prevalence (2.0%) was found among people 60 years and older, suggesting that systematic screening for HCV markers should be offered to this population through national screening campaigns, at primary care visits or at the time of hospital attendance [32], as this population has increased comorbidities and higher risk of hospitalisation. Furthermore, our survey indicates geographical heterogeneity in the prevalence of HCV infection, which requires regional adaptation of screening programmes to improve results and optimise the use of resources. The high level of HCV seroprevalence among people who inject drugs in Romania [33] warrants a complementary focus on key populations through accurately defining the HCV burden and scaling up efforts to diagnose infected individuals and link them to care. In addition, ongoing efforts to provide comprehensive prevention measures and improve access to treatment remain key pillars of hepatitis C elimination programmes. It is encouraging that the decreasing prevalence between previous studies and our current estimates provide an indication that Romania is on the right path towards the elimination of hepatitis C.

## Conclusion

The results of our study emphasise that Romania became a country with low prevalence of hepatitis C and is progressing towards ending the hepatitis C epidemic. Two population groups, those aged 60 years and older and those living in the North-East region of the country, should be prioritised for screening and intervention strategies.

## Supplementary Data

166000 Supplement
