# Supplementary material for: Prevalence of chronic hepatitis C infection in the general population: results from a national survey, Romania, 2020 to 2023
Source: Euro Surveill. 2024 Jul 25;29(30):2300663. doi: 10.2807/1560-7917.ES.2024.29.30.2300663 (PMC11274849; doi:10.2807/1560-7917.ES.2024.29.30.2300663)
Supplement: Supplement [file 23-00663_POPOVICI_Supplement.pdf]

This supplementary material is hosted by Eurosurveillance as supporting information alongside the article "Prevalence of chronic hepatitis C infection in the general population: results from a national survey, Romania, 2020 to 2023", on behalf of the authors, who remain responsible for the accuracy and appropriateness of the content. The same standards for ethics, copyright, attributions and permissions as for the article apply. Supplements are not edited by Eurosurveillance and the journal is not responsible for the maintenance of any links or email addresses provided therein.

Table 1. Demographic characteristics of the study sample and the general adult population in 2020 in Romania

Demographic characteristics		Total adult Romanian population* N=15,631,327		Study population (unweighted) N=2,674		Study population (weighted) N=2,674	
		N	%	N	%	N	%
**Sex**	Female	8,069,589	51.6%	1,427	53.4%	1,380.44	51.6%
	Male	7,561,738	48.4%	1,247	46.6%	1,293.56	48.4%
**Age groups (years)**	18-29	2,439,659	15.6%	384	14.4%	417.34	15.6%
	30-39	2,664,922	17.0%	382	14.6%	455.88	17.0%
	40-49	3,008,658	19.2%	382	14.8%	514.68	19.2%
	50-59	2,568,144	16.4%	379	14.9%	439.32	16.4%
	60-69	2,472,318	15.8%	379	15.2%	422.93	15.8%
	70-79	1,549,432	9.9%	386	15.8%	265.06	9.9%
	≥ 80	928,194	5.9%	382	16.0%	158.78	5.9%
**Region**	Bucuresti-Iflov	1,905,677	12.2%	332	12.4%	326.00	12.2%
	Center	1,853,820	11.9%	341	12.8%	317.13	11.9%
	North-East	2,484,551	15.9%	337	12.6%	425.02	15.9%
	North-West	2,056,887	13.2%	333	12.5%	351.86	13.2%
	South-East	1,921,357	12.3%	330	12.3%	328.68	12.3%
	South-Muntenia	2,369,689	15.2%	336	12.6%	405.37	15.2%
	South-West Oltenia	1,582,988	10.1%	330	12.3%	270.80	10.1%
	West	1,456,358	9.3%	335	12.5%	249.13	9.3%

* TEMPO Online [Internet]. [cited 2023 Sep 15]. Available from: http://statistici.insse.ro:8077/tempo-online/#/pages/tables/insse-table
